# Risk factors for child stunting in Bangladesh: an analysis using MICS 2019 data

**DOI:** 10.1186/s13690-022-00870-x

**Published:** 2022-04-21

**Authors:** Tuhinur Rahman Chowdhury, Sayan Chakrabarty, Muntaha Rakib, Stephen Winn, Jason Bennie

**Affiliations:** 1 Department of Agribusiness and Applied Economics, North Dakota State University, Fargo, ND 58105 USA; 2 Centre for Health Research, University of Southern Queensland, Springfield, QLD 4300 Australia; 3 Department of Economics, Shahjalal University of Science & Technology, Sylhet Kumargaon, Sylhet, 3114 Bangladesh; 4 School of Education, Edith Cowan University, Joondalup, Australia

**Keywords:** Child stunting, Parental education, Wealth index, Child undernutrition, Bangladesh

## Abstract

**Background:**

Malnutrition is considered a major public health challenge and is associated with a range of health issues, including childhood stunting. Stunting is a reliable and well-recognized indicator of chronic childhood malnutrition. The objective of this study is to determine the risk factors associated with stunting among 17,490 children below five years of age in Bangladesh.

**Methods:**

Correlates of child stunting were examined using data generated by a cross-sectional cluster survey conducted in Bangladesh in 2019. The data includes a total of 17,490 children (aged < 5 years) from 64,400 households. Multiple logistic regressions were used to determine the risk factors associated with child stunting and severe stunting.

**Results:**

The prevalence of stunting and severe stunting for children was 25.96% and 7.97%, respectively. Children aged 24 to < 36 months [Odds Ratio (OR) = 2.65, 95% CI: 2.30, 3.05] and aged 36 to < 48 months [OR = 2.33, 95% CI: 2.02, 2.69] had more risk of stunting compared to the children aged < 6 months. Children from Sylhet division had the greatest risk of stunting of all the eight divisions [OR = 1.26, 95% CI: 1.09, 1.46]. Children of secondary complete or higher educated mothers were less likely to develop stunting [OR = 0.66, 95% CI: 0.56, 0.79] compared with children of mothers having no education at all. Similarly, children of secondary complete or higher educated father [OR = 0.74, 95% CI: 0.63, 0.87] were found to have lower risk of stunting compared with children whose father hadn’t any education. Substantially lower risk of stunting was observed among children whose mother and father both completed secondary education or above [OR = 0.59, 95% CI: 0.52, 0.69]. Children from the richest households [OR = 0.49, 95% CI: 0.41, 0.58] had 51% lower odds of stunting compared to children from the poorest households.

**Conclusions:**

After controlling for socioeconomic and demographic factors, parental education and household position in the wealth index were found to be the most important determinants of child stunting in Bangladesh.

## Background

Malnutrition represents an insufficient intake of calories and nutrients that results in illness and in extreme cases, death [1]. Malnutrition among children poses a serious health threat to survival and it is linked to 3.1 million child deaths globally [2]. Among developing countries, it is estimated that approximately13 million children who are below five years of age die annually, of which malnutrition is among the leading causes [3, 4].

Height-for-age is one of the prominent parameters to investigate malnutrition status of a child [5]. According to the World Health Organisation (WHO), a child is regarded as stunted when his/her height < − 2 standard deviations from the child growth standard median for the same age and same sex [6]. Stunting represents a linear growth delay in a child. Globally, 159 million children are classified as stunted, indicating the prevalence rate of 23.8% [7]. Other negative health consequences of stunting among children include, a reduce recovery capacity from disease [8], increased risk of future adult obesity [9], poor cognitive functioning such as loss of memory, lower achievement in learning and increased attention deficit [10]. Stunting is estimated to be responsible for 1.2 million deaths globally for children under five years of age [2]. Malaria and pneumonia are more prevalent among children who are stunted and there is compelling evidence to support this [11, 12]. Stunting is also associated with unfavourable pregnancy outcome [13]. Stunting continues to be a hindrance to human capital development because of its negative impact on economic productivity of adults [14].

Recently, public health researchers have examined the risk factors associated with stunting. Most studies have shown that socioeconomic and demographic factors are key correlates of child stunting [15]. Among a sample of Nigerian children, low maternal education is found to be associated with a two-fold increase in risk for child stunning [16]. Socioeconomic status is another factor associated with child stunting. Children from a higher socioeconomic status have a significantly lower risk of stunting compared to children from a lower socioeconomic status [3]. Ricci [17] found that among Philippine children, a combination of factors, such as high socioeconomic status, being female, breastfeeding and more frequent prenatal care are important for reducing the risk of child stunting.

While these studies offer some insight into the factors associated with stunting across different countries, to date, little is known about how parental education and socio-economic status influence stunting among children five years old and below in Bangladesh. However, data suggest that Bangladesh is among the most child stunting prevalent countries in the world, with a stunting prevalence rate of 36.1%, which ranks it 107 out of 132 countries when the countries are ranked from lowest to highest for the prevalence of stunting [7]. Additionally, stunting is an impediment in the successful achievement of Sustainable Development Goals (SDGs) as it reduces the productivity of an individual and consequently negatively effects the likelihood of future economic growth. As one of the top performing countries in Millennium Development Goals (MDGs), Bangladesh is particularly keen to embrace the new SDGs targets. For example, reducing the proportion of stunting among children under five years of age from 36.1 to 25% is integrated in the 7th Five Year Plan of Bangladesh Government (2016–2020) to achieve the national SDG targets. Therefore, identifying the risk factors associated with child stunting in Bangladesh is a public health priority. In addition to stunting, severe stunting which is defined as height < − 3 standard deviations from child growth standard median for the same age and the same sex [6] is also in our interest since it is a measure of severe growth impairment for a child. Understanding risk factors for both stunting and severe stunting would help policy formulation to combat child chronic undernutrition problem. The aim of this study is to explore the risk factors that are associated with stunting and severe stunting among a large sample of children five years and below in Bangladesh.

## Data and method

Data was drawn from Multiple Indicators Cluster Survey (MICS) 2018–19 [18]. This survey was carried out by the Bangladesh Bureau of Statistics (BBS) with the support of the United Nations International Children’s Emergency Fund (UNICEF). The survey was conducted from January 19 to June 1, 2019, with the purpose of collecting data on important indicators at the national level for the eight divisions of the country. The survey sample was selected using a two-stage stratified cluster sampling approach, using the 2011 census frame to select clusters. Standardised questionnaires were used in all data collection [18]. The data include complete information on 64,400 households and the questionnaires were completed by parents of 23,099 children, five years of age and younger. To avoid counting households more than once and minimize recall bias, households with more than one child under five years of age, only the youngest child was selected for our study [19]. Our final sample is composed of 17,490 children below five years of age.

## Statistical analysis

Nutritional status of a child is examined using three anthropometric measures: height-for-age z scores, weight-for-age z scores and weight-for-height z scores [20]. Height-for-age z score is used to calculate stunting. There is a reference distribution of height for how children under the age of five should grow in a well-nourished population. Height-for-age can be expressed in standard deviation units (z-scores) from the median of the reference population. Multiple Indicator Cluster Survey 2019 (MICS) used WHO growth standard for reference population and calculating height-for-age z scores. We used MICS provided height-for-age z scores for creating dependent variable (stunting condition), as long-term growth of children can be reflected by height-for-age z scores, and it also can capture the effects of chronic malnutrition. Using WHO child growth standards as reference [21], the prevalence of childhood stunting in our sample was assessed using two classifications: (i) *Stunted*: defined as height-for-age z-scores less than − 2 standard deviations; and (ii) *Severely Stunted*: defined as children with height-for-age z-scores less than − 3 standard deviations. Before to begin the analysis, data cleaning was completed and checked for quality of data. There were no missing observations or Height-for-Age z scores that were unusually large or small.

We developed two separate models: (i) one to examine the associations of parental education and wealth with child stunting status (Table 2) and (ii) with child severe stunting status given other factors (Table 4). For the first model, the dependent variable was dichotomous (*Stunted* vs. *Not Stunted*), and for the second model, dependent variable was also dichotomous (*Severely Stunted* vs *Not Severely Stunted*) [22].

Independent variables included in the model are household member, age, gender, area, education of mother, education of father, wealth index quintile [19, 23], type of toilet facility [17], number of under five children in a household [23], salt iodization test outcome [24] and division [3]. To determine if there was any association between child stunting and the size of household, the household size was categorized in three categories based on the number of household members in each household (*up to three members*, *four to six members* and *seven or more*). This was included because family size is an important determinant of child malnutrition as caring for children and availability of food for consumption dependant on the number household members [25, 26]. Children’s ages were divided into six categories (*0 to < 6*, *6 to < 12*, *12 to < 24*, *24 to < 36*, *36 to < 48* and *48 to < 60*) with children less than six months (*0 to < 6*) as reference category. Children’s age was included in the model as it has been reported by many studies as a significant predictor of child stunting status [19, 27].

We included gender as an independent variable to examine if there was any significant difference of likelihood of stunting between male and female children. Administrative geography of Bangladesh is divided into 8 divisions, and we included division as a predictor variable in the regression model. Dhaka, Barisal, Chittagong, Khulna, Rajshahi, Rangpur, Sylhet, and Mymensingh were the eight divisions in Bangladesh where data was collected. Several studies on child under-nutrition in Bangladesh used geographical location (division) as a predictor of child stunting [3, 28]. Area (Urban/rural) variable was also included in the model. It is the most common variable included in almost all studies conducted in Bangladesh for identifying socioeconomic risk factors of malnourished children in Bangladesh [3, 19, 28, 29].

We included education of mother and education of father in our regression model to examine their role on the risk of child stunting and severe stunting. Parental education, both for education level of father and that of mother, was categorized into four categories based on the number of years of schooling: none for no schooling, primary schooling (*years 1 to < 6*), secondary incomplete (*years 6 to < 10*) and secondary complete or higher (≥*10*).

Wealth index was used as the indicator of socioeconomic status of households. Wealth index was constructed by principal components analysis in Bangladesh Multiple Indicators Cluster Survey 2019. In calculating wealth index, source of water, type of housing, type of toilet facility, type of fuels for cooking, electricity, bank account, some durable goods and animals were taken into consideration [18]. Households were assigned wealth score depending on the asset they owned. They used wealth scores of sampled households to rank them, and the households were then divided into five equal portions (quintiles) from poorest to wealthiest, such as poorest, second, middle, fourth and wealthiest. To assess the impact of latrine facilities on child stunting and severe stunting, we included type of toilet facility as predictor variable in our model. Iodine is an essential micronutrient and sufficient intake of iodine is necessary for normal growth [30]. Farebrother et.al [30]. also argued that sufficient intake of iodine prevents stunted growth and if the required daily minimum intake of iodine is not met, growth will be hampered. We therefore included salt iodization test outcome in our regression model to accommodate the role of iodized salt in reducing the risk of stunting among children who were aged five years and below. In multiple indicators cluster survey 2019, salt iodization test was carried out in each household and the result was classified into four categories namely, 0 PPM (*not iodized*), more than 0 PPM and less than 15 PPM, 15 PPM or more and no salt in the household. 15 PPM means salt containing 15 ppm (*PPM*) of iodate or iodide. In this survey, a cut-off point was set at 15 ppm indicating that salt containing 15 ppm or more of iodate or iodide will be considered as adequately iodized [18]. While selecting variables in our model, we considered variables used in previous studies and variables which could be relevant in the context of study in Bangladeshi population.

As our dependent variable in the first model was binary in nature (*Stunted* vs *Not Stunted*), we used binary logistic regression model to determine the relationship between socio-demographic factors and child stunting. Likewise, dependent variable in the second model was dichotomous (*Severely Stunted* vs *Not Severely Stunted*). Therefore, we also used a binary logistic regression model to determine the association between socio-demographic factors and child severe stunting. Nagelkerke R Square was estimated as 0.07 for the first model (χ^2^_(30)_ = 842; *p* < 0.001) and Nagelkerke R Square was estimated as 0.05 for the second model (χ^2^_(30)_ = 333; p < 0.001). We found no evidence for multicollinearity using a test for variance inflation factor (VIF) with estimated mean VIF of 1.72, implying that the level of multicollinearity is within tolerance limit [31].

## Results

### Descriptive characteristics of the sample

  Table 1 shows the socioeconomic and background characteristics of the sample and the bivariate distribution of stunting and severe stunting with sample characteristics. Data were available for 17,490 children aged < 5 years. A total of 25.96% children were estimated to be stunted, and 7.97% severely stunted. More than half of the children were male (52.53%). Children from Dhaka division comprised the highest proportion (18.93%) and the lowest percentage of study children were from Mymensingh division (6.11%). Most of the children (68.64%) were living in households with 4–6 family members. Approximately one quarter of mothers had not gone to school. However, the highest proportion of mothers (49.17%) had attended secondary school. An overwhelming majority of children (80.32%) came from rural areas. Nearly one quarter (21.93%) of children were aged between 12 and 35 months and 12.24% of children were less than six months of age. The highest percentage of children (26.18%) lived in poorest households and the lowest percentage of children (14.96%) lived in richest households in the category of wealth index quintile. 42.68% of children had access to flush toilet, whereas a majority (57.32%) of households had access to pit toilet or hanging latrine. Around 21% of fathers had not undertaken any formal education, however, approximately 16% of fathers were found to have completed secondary or higher secondary education.

**Table 1:** The bivariate distribution of stunting and severe stunting with sample characteristics using MICS 2019 data from Bangladesh

Characteristics	Total Sample (*n* = 16,597)	Stunted			Severely Stunted		
	%	Proportions (%)	95% CI	P- value	Proportions (%)	95% CI	*P* value
Household Members				0.163			0.069
<=3	14.16	0.2694 (26.94)	(0.2519907–0.2873285)		0.0775 (7.75)	(0.0673123–0.0887885)	
4–6	68.64	0.2554 (25.54)	(0.2475905–0.2632729)		0.0775 (7.75)	(0.0728217–0.0824734)	
> = 7	17.20	0.2686 (26.86)	(0.2528423–0.284843)		0.0901 (9.01)	(0.0800974–0.100894)	
Age (Months)				< 0.001			< 0.001
0 to < 6	12.24	0.1630 (16.30)	(0.1475978–0.1793465)		0.0621 (6.21)	(0.0522679–0.0731916)	
6 to < 12	10.87	0.1867 (18.67)	(0.1694561–0.2050035)		0.0631 (6.31)	(0.0526106–0.0750099)	
12 to < 24	21.93	0.2847 (28.47)	(0.2704294–0.2992424)		0.0905 (9.05)	(0.081566–0.0999811)	
24 to < 36	20.06	0.3229 (32.29)	(0.3074236–0.33864)		0.1037 (10.37)	(0.0938353–0.1142968)	
36 to < 48	18.78	0.2932 (29.32)	(0.2777072–0.3091417)		0.0816 (8.16)	(0.072468–0.0915005)	
48 to < 60	16.12	0.2302 (23.02)	(0.2147923–0.2462167)		0.0575 (5.75)	(0.0491617–0.0667055)	
Gender				< 0.001			0.271
Male	52.53	0.2646 (26.46)	(0.255586–0.2737319)		0.0818 (8.18)	(0.0763195–0.0876391)	
Female	47.47	0.2542 (25.42)	(0.2448175–0.2636666)		0.0773 (7.73)	(0.0716738–0.0832865)	
Area				< 0.001			0.071
Urban	19.68	0.2275 (22.75)	(0.2135702–0.2418622)		0.0723 (7.23)	(0.0639082–0.0815099)	
Rural	80.32	0.2675 (26.75)	(0.2602045–0.2749147)		0.0815 (8.15)	(0.0770331–0.0861536)	
Division				< 0.001			< 0.001
Barishal	9.35	0.2667 (26.67)	(0.245167–0.2753425)		0.0862 (8.62)	(0.0730781–0.1009056)	
Chattogram	18.20	0.2545 (25.45)	(0.2453603–0.2888139)		0.0798 (7.98)	(0.0706173–0.0897611)	
Dhaka	18.93	0.2600 (26.00)	(0.2394194–0.2699869)		0.0876 (8.76)	(0.0781742–0.097735)	
Khulna	14.86	0.2001 (20.01)	(0.1848487–0.2159838)		0.0385 (3.85)	(0.0314134–0.0466015)	
Mymenshing	6.11	0.3099 (30.99)	(0.2822769–0.3386249)		0.0908 (9.08)	(0.074268–0.1096732)	
Rajshahi	11.64	0.2509 (25.06)	(0.2319099–0.2700397)		0.0649 (6.49)	(0.0545517–0.0764521)	
Rangpur	13.39	0.2656 (26.56)	(0.2477871–0.283971)		0.1008 (10.08)	(0.0888687–0.1136772)	
Sylhet	7.53	0.3424 (34.24)	(0.3168128–0.3687823)		0.1093 (10.93)	(0.0929921–0.1274525)	
Education of Mother				< 0.001			< 0.001
Pre-primary or none	10.89	0.3682 (36.82)	(0.3464627–0.3902887)		0.1250 (12.50)	(0.1104685–0.1406971)	
Primary	24.87	0.3097 (30.97)	(0.2960008–0.3237079)		0.1005 (10.05)	(0.0917032–0.1098031)	
Secondary	49.17	0.2364 (23.64)	(0.2274482–0.2455214)		0.0671 (6.71)	(0.0618947–0.072587)	
Higher secondary+	15.08	0.1744 (17.44)	(0.1601345–0.1894734)		0.0538 (5.38)	(0.0455437–0.0631615)	
Education of Father				< 0.001			< 0.001
Pre-primary or none	20.80	0.3340 (33.40)	(0.3186501–0.3495665)		0.1072 (10.72)	(0.0973299–0.1177096)	
Primary	31.98	0.2866 (28.66)	(0.2747327–0.2986043)		0.0887 (8.87)	(0.0813439–0.0964212)	
Secondary	31.18	0.2281 (22.81)	(0.2170497–0.2395045)		0.0629 (6.29)	(0.0566008–0.0696741)	
Higher secondary+	16.04	0.1708 (17.08)	(0.1570114–0.1852121)		0.0588 (5.88)	(0.0504019 0.0681796)	
Wealth Index quintile				< 0.001			< 0.001
Poorest	26.18	0.3405 (34.05)	(0.3267383–0.3544001)		0.1103 (11.03)	(0.1013546–0.1197179)	
Second	22.37	0.2835 (28.35)	(0.2694019–0.2978956)		0.0851 (8.51)	(0.0765624–0.0943085)	
Middle	18.87	0.2352 (23.52)	(0.2207703–0.2500036)		0.0655 (6.55)	(0.0572527–0.0744363)	
Fourth	17.62	0.2112 (21.12)	(0.196933–0.2260701)		0.0607 (6.07)	(0.0525045–0.0696908)	
Richest	14.96	0.1704 (17.04)	(0.156202–0.1853876)		0.0585 (5.85)	(0.049782–.0681491)	
Type of toilet facility				< 0.001			< 0.001
Flush latrine	42.68	0.2170 (21.70)	(0.2077067–0.2265402)		0.0682 (6.82)	(0.2077067–0.2265402)	
Pit and hanging latrine	57.32	0.2914 (29.14)	(0.2824885–0.300376)		0.0883 (8.83)	(0.2824885–0.300376)	
Salt Iodization test outcome				< 0.001			< 0.001
0 PPM (not iodized)	25.73	0.2835 (28.35)	(0.2703587–0.2969078)		0.0904 (9.04)	(0.0822027–0.0991818)	
More than 0 PPM and less than 15 PPM	19.01	0.2819 (28.19)	(0.2666411–0.2975221)		0.0890 (8.90)	(0.0795813–0.0992474)	
15 PPM or more	54.75	0.2399 (23.99)	(0.231346–9.2485535)		0.0713 (7.13)	(0.0662482–0.0766627)	
No salt in the household	0.51	0.3483 (34.83)	(0.2503611–0.4566767)		0.0899 (8.99)	(0.03961–0.1694525)	
Stunting condition							
Stunted (HAZ score < −2 SD)	25.96						
Severely stunted (HAZ < −3 SD)	7.97						

### Risk factors for child stunting

Similar proportions of male children (26.46%) and female children (25.42%) were classified as stunted (Table 1). The prevalence rate of stunting was highest for those children whose parents had no education and whose family used a hanging toilet. Half of the children classified as stunted were aged between 24 and 35 months. The largest proportion of stunted children came from Sylhet division (34.24%) and the lowest proportion from Khulna division (20.01%).

In bivariate analysis, the following variables were significantly associated with stunting prevalence: age (*p* < 0.001), gender (p < 0.001), area (p < 0.001), division (p < 0.001), education of mother (p < 0.001), education of father (p < 0.001), wealth index quintile (p < 0.001), type of toilet facility (p < 0.001) and salt iodization test outcome (p < 0.001).

Table 2 shows the results for binary logistic regression analysis on the risk factors associated with child stunting. Children’s age was a significant predictor of stunting. Those aged twelve months to less than twenty-four months had significantly higher odds of stunting [OR: 2.16, 95% CI: 1.88–2.48] compared to children below six months of age. Children aged twenty-four to less than thirty-six months had almost three-fold odds of stunting [OR: 2.65, 95% CI: 2.30–3.05] compared to the children aged less than six months. Children living in Khulna division were significantly less likely to be stunted [OR: 0.71, 95% CI: 0.62–0.81] compared with the children who lived in Dhaka division. Children from Sylhet division had significantly higher odds of stunting compared with children from Dhaka division [OR: 1.26, 95% CI: 1.09–1.46]. Across categories of parental education, children whose mothers had completed secondary level education or higher were less likely to be stunted in comparison with the children whose mothers had no formal education [OR: 0.66, 95% CI: 0.56–0.79]. There were significantly lower odds of stunting for the children whose fathers had completed secondary education or higher [OR: 0.74, 95% CI: 0.63–0.87] compared to those children whose father had no formal education. Children from households with middle category in wealth index quintile were less likely to be stunted [OR: 0.69, 95% CI: 0.62–0.77] than the children from households with poorest category. Children of wealthiest families had 59% lower odds of stunting [OR: 0.49, 95% CI: 0.41–0.58] compared to those from poorest families. Children from families which used hanging toilet had larger odds of stunting [OR: 1.21, 95% CI: 1.03–1.41] compared with those from those families that used flush toilet.

**Table 2:** Risk factors for stunting (HAZ scores < −2SD) among children aged less than five years using MICS 2019 data from Bangladesh

Factors		OR	95% CI
Household member	≤3	**ref**	
	4--6	0.90	[0.81–1.00]**
	≥7	0.98	[0.86–1.11]
Age (Months)	0 to < 6	**ref**	
	6 to < 12	1.23	[1.04–1.45]**
	12 to < 24	2.16	[1.88–2.48]***
	24 to < 36	2.65	[2.30–3.05]***
	36 to < 48	2.33	[2.02–2.69]***
	48 to < 60	1.67	[1.44–1.95]***
Gender	Male	**ref**	
	Female	0.94	[0.88–1.01]
Area	Urban	**ref**	
	Rural	0.90	[0.81–1.00]**
Division	Dhaka	**ref**	
	Barishal	0.87	[0.76–1.01]*
	Chattogram	0.87	[0.77–0.97]**
	Khulna	0.71	[0.62–0.81]***
	Mymenshing	1.06	[0.91–1.25]
	Rajshahi	0.89	[0.78–1.01]*
	Rangpur	0.90	[0.79–1.02]
	Sylhet	1.26	[1.09–1.46]***
Education of mother (schooling years)	None (0)	**ref**	
	Primary (1 to < 6)	0.87	[0.77–0.99]**
	Secondary incomplete (6 to < 10)	0.74	[0.65–0.83]***
	Secondary complete or higher (≥10)	0.66	[0.56–0.79]***
Education of father (schooling years)	None (0)	**ref**	
	Primary (1 to < 6)	0.95	[0.86–1.04]
	Secondary incomplete (6 to < 10)	0.83	[0.75–0.93]***
	Secondary complete or higher (≥10)	0.74	[0.63–0.87]***
Wealth index quintile	Poorest	**ref**	
	Second	0.83	[0.75–0.91]***
	Middle	0.69	[0.62–0.77]***
	Fourth	0.63	[0.55–0.72]***
	Richest	0.49	[0.41–0.58]***
Toilet facility	Flush latrine	**ref**	
	Pitn and hanging latrine	0.99	[0.91–1.09]
Salt idolization test	0 PPM (not iodized)	**ref**	
	More than 0 PPM and less than 15 PPM	1.01	[0.91–1.12]
	15 PPM or more	0.98	[0.90–1.07]
	No salt in the household	1.52	[0.97–2.40]*
Number of under five children		1.25	[1.14–1.37]***

*** p < 0.001, ** *p* < 0.01, * *p* < 0.05

Table 3 shows the effects of parental combined level of education on the risk of child stunting after adjustment for household member, age, gender, area, division, wealth index, type of toilet facility, salt iodization test outcome and number of under five children. The results suggest that one parent with primary school and one with secondary school and above [OR: 0.73, 95% CI: 0.63–0.84] and both parents with secondary school and above [OR: 0.59, 95% CI: 0.52–0.69] had significantly lower likelihood of stunting among their children when compared with children of parents who both had no education.

**Table 3:** Effects of parental combined level of education on the risk of stunting using MICS 2019 data from Bangladesh

Level of Education	OR	95% CI
Level 1	**Ref.**	
Level 2	0.94	[0.81–1.10]
Level 3	0.78	[0.66–0.93]***
Level 4	0.84	[0.72–0.97]**
Level 5	0.73	[0.63–0.84]***
Level 6	0.59	[0.52–0.69]***

*** p < 0.001, ** p < 0.01, * p < 0.05

Level 1 = both parents no education; Level 2 = one with no education, one with primary school; Level 3 = one with no education, one with secondary school and above; Level 4 = both with primary school; Level 5 = one with primary school, one with secondary school and above; and Level 6 = both with secondary school and above [40]

### Risk factors for severe stunting

Table 1 shows the proportion of children classified as severely stunted. The percentage of male children who were severely stunted (8.18%) was about the same as the percentage of female children who were severely stunted (7.73%). A slightly higher percentage of rural children were severely stunted (8.15%) compared with the urban children (7.23%). The highest proportion of severely stunted children was found in the Sylhet division (10.93%) and the lowest in the Khulna division (3.85%). Approximately one-tenth of children, with their mothers had no education were severely stunted (10.93%), and nearly 6% were severely stunted if their mothers completed secondary education or higher. Similarly, only 5.88% of children were severely stunted whose fathers had completed secondary or higher education and more than one-tenth (10.72%) of children were severely stunted whose father didn’t go to school. Only 5.85% of children from wealthiest families were severely stunted and 11.03% of children from poorest families were severely stunted. The highest rate of severe stunting prevalence was found for children age between 24 and 35 months (10.37%) and the lowest rate for age less than six months (6.21%).

In bivariate analysis, the following variables were significantly associated with the prevalence of severe stunting: age (*p* < 0.001), division (p < 0.001), education of mother (p < 0.001), education of father (p < 0.001), wealth index (p < 0.001), type of toilet facility (p < 0.001) and salt iodization test outcome (p < 0.001). The results on the binary logistic regression analysis on the risk factors that were associated with the risk for severe stunting among the children five years and below can be found in Appendix Table 4. Children aged twenty-four to less than thirty-six months were almost two times more likely to be severely stunted [OR: 1.85, 95% CI: 2.37–3.52] compared with the children below six months of age. There was 44% higher the risk of severe stunting for children who were aged thirty-six to less than forty-eight months [OR: 1.44, 95% CI: 1.15–1.80] compared to those who were less than six months of age. Children whose mothers’ education level was secondary incomplete [OR: 0.68, 95% CI: 0.57–0.82] or secondary complete or higher [OR: 0.59, 95% CI: 0.44–0.77] were significantly less likely to be severely stunted compared with the children whose mothers had no education. Moreover, children whose fathers had education level secondary incomplete, had 19% lower odds that they could be severely stunted. Children had 57% lower odds to be severely stunted if they lived in Khulna division [OR: 0.43, 95% CI:0.34–0.54] compared with the children who lived in Dhaka division. Across categories of wealth, those who were in the fourth category in wealth index quintile, had 37% lower odds of severe stunting compared to children from poorest families. Children from wealthiest families had 44% lower odds of severe stunting than the children who lived in poorest families.

The results from A2 suggests that after adjustment for: household member, age, gender, area, division, wealth index, type of toilet facility, salt iodization test outcome and number of under five children in a household, any increase in the level of parental education are associated with lower odds of severe stunting among children below 5 years (Appendix Table 5).

## Discussion

This study examined the prevalence rate of stunting among a large sample of Bangladeshi children aged < 5 years. Our data showed that national prevalence rate for child stunting is at 25.96%. This prevalence is remarkably lower than the 41% observed in 2007 [32], suggesting that child stunting in Bangladesh has declined over time.

A key finding was that our adjusted analysis showed that children living in Sylhet division had the highest risk for being stunted. Food security status in this region has been suggested to be relatively better than the other divisions of Bangladesh [32]. Furthermore, the rate of poverty is lower in Sylhet division compared with other divisions in Bangladesh [33]. Poor educational opportunities and access, adverse maternal health [34] might explain why children from Sylhet division had high odds of child stunting. Parental education has consistently been shown to protect children from stunting. Sylhet division, on the other hand, has the worst educational standing of any Bangladeshi division. Sylhet has the second lowest primary completion rate in the country at 78%. Additionally, it has the lowest completion rates for lower secondary and upper secondary education, with 53 and 22%, respectively [35]. In Bangladesh, mothers generally spend more time with children than fathers, and Sylhet division trails behind other divisions in terms of female education. Thus, the current nutritional situation can be partially attributed to a lack of education in Sylhet division. Education and specifically access to secondary education is and remains a major issue for Bangladesh in general, and for specific Divisions within Bangladesh. The analysis required is beyond the scope of this paper but will be the focus of a future paper that examines if there is any correlation between access to school education, parental income, and nutrition.

Even though Sylhet is suggested to have better economic condition than any other region of the country, it is important to note that there are still inequalities in this region. For example, the Sylhet region is geographically heterogeneous with tea gardens, hills and haors (a haor is a marshy wetland ecosystem in the north-eastern part of Bangladesh that looks like inland seas during the monsoon floods). In those areas transport facilities are poor which hinders students to attend schools and hence there are few opportunities for educational attainment by children. Our findings suggest that future research is needed to determine the factors associated with the high prevalence rate of child stunting in the Sylhet region.

A further key finding of the present study was that wealth index was a significant protective factor against child stunting. This finding is consistent with other studies showing that household socioeconomic status was a significant predictor of child stunting [36–42]. A study conducted by Talukder, reported that Bangladeshi children living in the poorest household had 38% higher odds for malnourishment compared to the children from the wealthiest family [43]. The lower odds of child stunting associated with higher wealth index might be explained by the fact that children from wealthier families have better access to health facilities, better environmental conditions such as potable water, sanitation, and access to sufficient food. It might also be possible that parents from wealthiest families have a higher education and are more responsible for the health of their children when compared with parents from the poorest households. Other studies have shown that low quality housing and no access to potable water were significantly associated with child stunting [44, 45]. According to UNICEF (1990), major determinants of nutritional condition for a child are access to sufficient food supplies, improved health facilities and access to safe potable water supplies that are determined by household economic status [43]. The key findings from the present study suggest that improvement in household wealth status can possibly have significant impact on reducing the risk of child stunting.

Consistent with other studies, we showed that maternal education has significant impact in reducing the chance for a child to be stunted [3, 6, 29, 37, 46, 47]. The presence of an educated mother might lead to a better understanding of nutritional conditions for her children. Better child feeding practices, for instance, exclusive breast feeding during first six months of a newborn child and timely initiation of complementary foods for children have been identified to be significantly associated with lower risk of child stunting [48]. More educated mothers may earn more money and therefore they may have more opportunity to invest in the health of their children [49]. Moreover, educated mothers are more likely to make better use of health care for their children [50, 51], to make efficient use of family resources [19, 52] and more willing to utilise family planning [16].

A further outcome from the present study was that a child’s age had a significant impact on child stunting. The chances of a child being stunted increased with age and reached a peak at 24–35 months. The finding is supported by other studies [52–54]. A potential cause of this might be that as the age of a child increases, biological factors and socioeconomic factors become greater determinants of stunting. It has been reported that male children had the higher chance of being stunted compared with female children [55, 56]. However, our study failed to establish significant association by gender for increased odds of stunting or severe stunting.

Children from households where salt was found to be adequately iodized (*≥15 PPM in salt iodization test*) had significantly lower odds of stunting, compared with the children from households where salt was found as not iodized (*0 PPM*). The role of iodized salt as an important protective factor against stunting was supported by other studies which also suggest iodized salt reduces the risk of stunting [24, 57]. Research has shown that iodine is crucial for normal physical growth [30] and insufficient intake of iodine by children during infancy and childhood could cause impaired growth [58, 59].

There are some limitations in the study that should be acknowledged. First, the cross-sectional design limits the possibility of determining causal relationships. Moreover, many studies have shown a seasonal impact on the prevalence of stunting especially in the rural areas of developing countries [60, 61]. Given our study design, we were not able to assess seasonal impact. A further limitation was that there was no information on childbirth weight, paternal height, maternal height, and maternal age, all of which have been reported as significant predictor of child malnutrition [17, 19]. Strengths of the study include the use of a large representative sample size with a standardized questionnaire. This allows for comparisons with future data collected as a part of the Bangladeshi Multiple Indicators Cluster Survey.

## Conclusion

Among a large sample of Bangladeshi children aged < 5 years, about 25.96% were classified as stunted. Key risk factors for child stunting included increased age, living in Sylhet division and more under five children in a household, using a pit toilet or a hanging toilet. In contrast, protective factors against child stunting were higher level of parental education and living in higher wealth index. Importantly, the risk factors as well as protective factors were similar between stunting and severe stunting implying intervention targeting the reduction of child stunting could eventually reduce the prevalence of severe stunting among children below five years of age. Our study suggested that parental education is a major protective factor that plays a crucial role in reducing the risk for a child to be stunted or severely stunted. Consequently, we recommend promotion of education both for men and women. Higher level of maternal education would improve child nutritional conditions through its role on influencing child feeding and childcare.

Promotion of parental education would improve child nutritional condition primarily through a generation of higher income and living in better neighbourhoods, that is living in areas where there are greater medical facilities and cleaner environment. Another crucial protective factor indicated by the study is socioeconomic status measured by the wealth index quintile. As regional differences appeared as a significant predictor of child stunting and severe stunting, for instance children living in Sylhet division are in higher risk for being stunted and severely stunted, geographical targeting should be adopted by governments and stakeholders on reducing stunting prevalence.

To reduce stunting prevalence in Bangladesh, policies should be implemented that focus on the risk factors determined by the study. Interventions regarding parental education and reducing socio-economic inequality may have long lasting beneficial effects on child malnutrition.

## Data Availability

The analysis was based on the Bangladesh Multiple Indicators Cluster Survey (MICS)- 2019 data, collected by Bangladesh Bureau of Statistics along with UNICEF who provided technical and financial support, data is available upon request at https://mics.unicef.org/surveys

## Appendix

**Table 4:** Risk factors for severe stunting (HAZ scores < −3SD) among children aged less than five years using MICS 2019 data from Bangladesh

Factors		OR	95% CI
Household member	≤3	**ref**	
	4--6	0.95	[0.80–1.12]
	≥7	1.09	[0.89–1.34]
Age (Months)	0 to < 6	**ref**	
	6 to < 12	1.06	[0.82–1.37]
	12 to < 24	1.56	[1.26–1.93]***
	24 to < 36	1.85	[1.50–2.29]***
	36 to < 48	1.44	[1.15–1.80]***
	48 to < 60	0.99	[0.77–1.26]
Gender	Male	**ref**	
	Female	0.94	[0.84–1.05]
Area	Urban	**ref**	
	Rural	0.89	[0.76–1.05]
Division	Dhaka	**ref**	
	Barishal	0.88	[0.71–1.10]
	Chattogram	0.82	[0.69–0.99]**
	Khulna	0.43	[0.34–0.54]***
	Mymenshing	0.88	[0.69–1.12]
	Rajshahi	0.70	[0.56–0.87]***
	Rangpur	1.06	[0.88–1.28]
	Sylhet	1.08	[0.87–1.34]
Education of mother (schooling years)	None (0)	**ref**	
	Primary (1 to < 6)	0.88	[0.74–1.06]
	Secondary incomplete (6 to < 10)	0.68	[0.57–0.82]***
	Secondary complete or higher (≥10)	0.59	[0.44–0.77]***
Education of father (schooling years)	None (0)	**ref**	
	Primary (1 to < 6)	0.97	[0.83–1.13]
	Secondary incomplete (6 to < 10)	0.81	[0.68–0.97]**
	Secondary complete or higher (≥10)	0.96	[0.75–1.24]
Wealth index quintile	Poorest	**ref**	
	Second	0.83	[0.71–0.97]**
	Middle	0.67	[0.56–0.81]***
	Fourth	0.63	[0.51–0.78]***
	Richest	0.56	[0.43–0.74]***
Toilet facility	Flush latrine	**ref**	
	Pitn and hanging latrine	0.87	[0.75–1.00]*
Salt idolization test	0 PPM (not iodized)	**ref**	
	More than 0 PPM and less than 15 PPM	0.97	[0.82–1.14]
	15 PPM or more	0.89	[0.77–1.02]
	No salt in the household	1.13	[0.54–2.39]
Number of under five children		1.19	[1.03–1.37]**

*** *p* < 0.001, ** *p* < 0.01, * *p* < 0.05

**Table 5:** Effects of parental combined level of education on the risk of severe stunting using MICS 2019 data from Bangladesh

Level of education	OR	95% CI
Level 1	**Ref.**	
Level 2	1.14	[0.91–1.43]
Level 3	0.89	[0.69–1.16]***
Level 4	0.93	[0.74–1.17]**
Level 5	0.74	[0.60–0.92]***
Level 6	0.63	[0.50–0.78]***

*** *p* < 0.001, ** *p* < 0.01, * *p* < 0.05

Level 1 = both parents no education; Level 2 = one with no education, one with primary school; Level 3 = one with no education, one with secondary school and above; Level 4 = both with primary school; Level 5 = one with primary school, one with secondary school and above; and Level 6 = both with secondary school and above [19]

## Abbreviations

BBS: Bangladesh Bureau of Statistics
CI: Confidence Interval
HAZ: Height for Age Z Score
MDGs: Millennium Development Goals
OR: Odds Ratio
PPM: Parts Per Million
SD: Standard Deviation
SDGs: Sustainable Development Goals
UNICEF: United Nations International Children’s Emergency Fund
VIF: Variance Inflation Factor
WHO: World Health Organization
